# Skin graft bolstered by negative pressure therapy in chronic wounds: A systematic review

**DOI:** 10.1111/jdv.20808

**Published:** 2025-08-27

**Authors:** M. Gamel, M. Gael, A. C. Bursztejn

**Affiliations:** ^1^ Dermatology, Batiment P Canton Centre Hospitalier Universitaire de Nancy Vandoeuvre les Nancy France

**Keywords:** chronic wounds, negative pressure wound therapy, skin graft

## Abstract

**Background:**

Chronic ulcers represent a significant burden for patients, often requiring advanced therapeutic interventions. Skin grafting is a critical treatment option, yet the risk of graft failure remains substantial. Negative pressure wound therapy (NPWT) has demonstrated efficacy in enhancing graft survival in acute wounds, but its effectiveness in chronic ulcers remains underexplored.

**Objectives:**

This systematic review aimed to evaluate the efficacy of NPWT in bolstering skin grafts for the treatment of chronic ulcers.

**Methods:**

We conducted this systematic literature review using the scientific databases PUBMED, COCHRANE and EMBASE. Studies involving skin grafts supported by NPWT for chronic wounds were included, while those focussing on acute wounds, burns or post‐surgical wounds were excluded. Data on patient demographics, wound characteristics, NPWT parameters and graft outcomes were extracted and analysed.

**Results:**

A total of 21 articles, encompassing 186 patients, were included. The maximal graft uptake (≥95%) was observed in 33% of patients, while 77% achieved a graft uptake of ≥90%. The mean graft take was 92% (89.7, 93.5, 95% confidence interval). Complete recovery was achieved in 79% of patients, with a mean recovery time of 73 days. Complete response without recurrence was noticed in 38% of the cases, with a mean time without recurrence of 197 days. Exploratory analyses indicated higher graft take rates in patients under 65 years, males, diabetics and those without arterial disease. Venous and neurotrophic ulcers also showed superior graft uptake.

**Conclusions:**

NPWT appears to significantly enhance graft survival in chronic ulcers, with an excellent safety and tolerance profile. As there was no control group, historical controls of graft take without NPWT were used. Short‐term NPWT application (less than 1 week) is recommended for optimal outcomes. These findings suggest that NPWT should be considered a preferred method for securing skin grafts in chronic ulcer management.

PROSPERO registration number: CRD420251005192.

## Graphical abstract

NPWT improve the graft take rate of skin graft in chronic ulcer, with an excellent safety and tolerance profile. We have identified various subgroups in which NPWT appears to be more effective, such as venous ulcers, men, patients aged under 65, without arterial disease and those with prior antibiotic therapy.

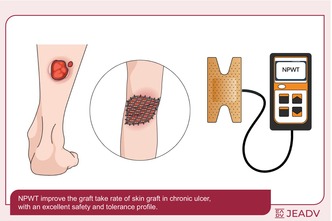

**Why was the study undertaken?**

- To evaluate the efficacy of NPWT in enhancing skin graft survival for chronic ulcers.

**What does this study add?**

- This study demonstrates that NPWT significantly improves graft uptake in chronic ulcers, with higher success rates in specific patient subgroups, such as those with venous ulcers, males and patients under 65 years of age. Short‐term NPWT application is most effective.

**What are the implications of this study for disease understanding and/or clinical care?**

- The use of NPWT in chronic ulcer management may lead to faster healing, reduced treatment costs and improved patient outcomes. Further large‐scale studies are needed to confirm these findings and identify optimal patient profiles for NPWT.

## INTRODUCTION

Chronic ulcers, defined as persistent skin defects lasting more than 1 month,^1^ represent a significant clinical challenge. Leg ulcers are a common condition, with an estimated prevalence between 0.5% and 1% of the general population and 3% of people aged over 65 years.^2^ The socio‐economic impact of chronic ulcers is substantial,^3^ driven by prolonged treatment durations, frequent hospitalizations, time off work^4^ and high recurrence rates. It also has a major impact on quality of life, particularly in terms of pain and the resulting functional disability. Despite advances in wound care, achieving complete healing remains elusive for many patients. The rate of ulcer recurrence is high, with over 50%–60% of venous ulcers recurring within a year.^5^, ^6^

Skin grafting is a well‐established treatment for chronic ulcers, particularly when conventional therapies fail. However, graft failure rates exceeding 30%^7^ underscore the need for adjunctive therapies to enhance graft survival. Negative pressure wound therapy (NPWT) has emerged as a promising intervention, demonstrating efficacy in acute wound settings such as surgery, burns or trauma.^8^, ^9^, ^10^, ^11^, ^12^ However, its role in chronic ulcer management remains underexplored. This systematic review aimed to evaluate the efficacy of NPWT in bolstering skin grafts for chronic ulcers.

## MATERIALS AND METHODS

### Search strategy

We conducted a systematic literature review in accordance with the Preferred Items for Systematic Review and Meta‐analysis (PRISMA) guidelines by searching the scientific databases PUBMED, COCHRANE and EMBASE with the following keywords and their synonyms in the titles and abstracts: “SKIN GRAFTS” and “NEGATIVE PRESSURE THERAPY.” The search equations used in each database are described in Appendices S1–S3 in Data S1. The search was performed from 01 January 1996 to 01 April 2023. The literature search was limited to articles on human subjects written in English, French and German. In addition, other publications were selected from the references of articles identified in the PUBMED, EMBASE and COCHRANE search. The results of the additional search were checked for duplicates and selected for inclusion.

The selection process consisted of reading titles and abstracts in order to retain only those articles dealing with skin grafting and negative pressure therapy as the main subject.

To assess eligibility, the full text of the selected articles was read.

### Inclusion and exclusion criteria

Studies involving skin grafts supported by NPWT for chronic wounds were included. Articles focussing on acute wounds, burns or post‐surgical wounds were excluded. Reviews, conference abstracts and studies with incomplete data on graft uptake were also excluded.

### Data extraction

Data on patient demographics (gender, age and comorbidities), wound characteristics (type of wound, size, location, duration, previous treatments, time to full recovery, relapse and duration without recurrence), NPWT parameters (NPWT type, pressure set, type of foam, duration, first opening, pace of change and side effects) and graft outcomes (graft type, graft take) were extracted.

### Primary and secondary endpoints

The primary outcome was maximal graft uptake (≥95%). Secondary outcomes included graft uptake ≥90%, time to complete recovery, proportion of patients with complete response without recurrence and proportion of patients who discontinued NPWT due to inefficacy or intolerance.

### Statistical analysis

Demographic and clinical characteristics are presented using percentage for categorical variables and mean standard deviation (SD) or median interquartile (IQR) for continuous variables.

Univariate logistic regression analyses were conducted to explore associations between graft take with NPWT and the following potential predictive factors: gender, age, aetiology, size, duration of the wound, prior NPWT and duration of NPWT on the graft.

The Chi2 test or Fisher's exact test were used for categorical variables where appropriate. Welsh's *t*‐test, the Mann–Whitney *U* test or the Kruskal–Wallis test were used for continuous variables where appropriate. Pearson's or Spearman's correlation coefficient tests were used to explore the direction of associations. In all cases, *p* < 0.05 was considered statistically significant. The analysis was performed using Medistica. *p*value.io (2019), a user interface for the statistical analysis software R. Missing values of key variables were imputed using the Multiple Imputation by Chained Equations (MICE) when a parameter had more than 5% missing data, and medians or modes when a parameter had less than 5% missing data.

## RESULTS

**FIGURE 1:**
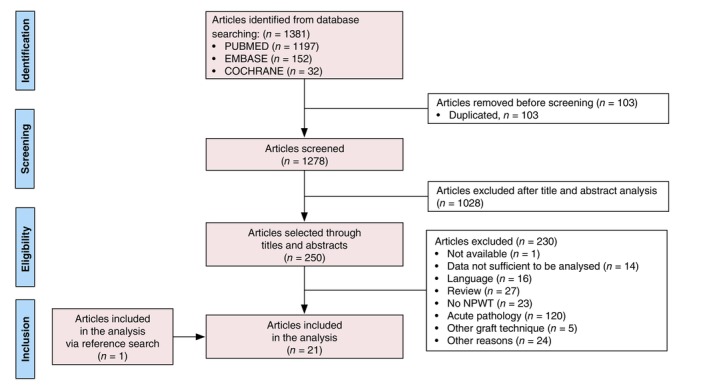
Flow chart.

A total of 1381 records were identified through database searches. Following the application of selection criteria, 1131 records were excluded, leaving 250 potentially relevant studies. Subsequently, 230 studies were excluded for various reasons, as illustrated in the search flow chart (Figure 1). Ultimately, 21 articles were included in the analysis, encompassing a total of 186 patients.^7^, ^12^, ^13^, ^14^, ^15^, ^16^, ^17^, ^18^, ^19^, ^20^, ^21^, ^22^, ^23^, ^24^, ^25^, ^26^, ^27^, ^28^, ^29^, ^30^, ^31^

In terms of study design, 12 articles were clinical case reports, four were case series, two were retrospective comparative studies, and three were prospective randomized controlled trials. The demographic and clinical characteristics of the patients are summarized in Table 1. The majority of the cohort was female (57%), with a mean age of 66.1 years. The most prevalent ulcer types were venous ulcers (54%), pyoderma gangrenosum (19%) and arterial ulcers (9.7%). Other ulcer types included neurotrophic ulcers (6.5%), mixed arterial and venous ulcers (3.2%), Martorell's ulcers (2.7%), calciphylaxis (1.6%), vasculitis (1.6%) and pressure sores (1.1%). The ulcers were predominantly located on the lower limbs (98%), with one pressure sore on the sacral area, one pyoderma gangrenosum on the occiput and one neurotrophic ulcer on the plantar surface of the foot. All wounds measured more than 10 cm^2^, with a mean size of 165 cm^2^. A triggering factor was reported in only 26% of cases, including minor trauma for Martorell's ulcers, prolonged immobility for pressure sores and phlebitis for venous ulcers.

**TABLE 1:** Patients' characteristics (*n* = 186).

		*n*
Sex M (%): F (%)	67 (43): 90 (57)	157
Age (year), mean [IC 95%], (min–max)	66.1 [64.2, 68], (16–89)	157
Age ≥ 65 years, *n* (%)	99 (63)	157
Wound type		
Venous ulcer, *n* (%)	101 (54)	186
Pressure sore, *n* (%)	2 (1.1)	186
Calciphylaxis, *n* (%)	3 (1.6)	186
Martorell's ulcer, *n* (%)	5 (2.7)	186
Pyoderma gangrenosum, *n* (%)	36 (19)	186
Neurotrophic ulcer, *n* (%)	12 (6.5)	186
Arterial ulcer, *n* (%)	18 (9.7)	186
Mixt arterial and venous ulcer, *n* (%)	6 (3.2)	186
Other (vasculitis), *n* (%)	3 (1.6)	186
Localization on leg, *n* (%)	183 (98)	186
Size (cm^2^), mean [IC 95%], (min–max)	165 [163.1, 166.9], (20–875)	139
Trigger factor^a^, *n* (%)	8 (26)	31
Venous disease, *n* (%)	83 (70)	118
Arterial disease, *n* (%)	23 (21)	109
Diabetes, *n* (%)	34 (23)	150
Positive skin sample^b^, *n* (%)	77 (53)	145
Wound duration (months), mean [IC 95%], (min–max)	27.01 [25.11, 28.91], (1–360)	167
Wound duration > 1 year, *n* (%)	71 (43)	167
Prior skin grafting, *n* (%)	3 (1.6)	186
Prior NPWT, *n* (%)	100 (54)	186
Duration of prior NPWT (days), mean [IC 95%], (min–max)	6.8 [4.9, 8.7], (2–35)	81
Antibiotics prior graft^c^, *n* (%)	51 (28)	183
Debridement prior graft, *n* (%)	95 (51)	186
Duration NPWT on graft (days), mean [IC 95%], (min–max)	6.14 [4.2, 8.0], (3–21)	166
Pressure set NPWT (mmHg), mean [IC 95%], (min–max)	104 [102.1, 105.9], (60–125)	135
First opening (days), mean [IC 95%], (min–max)	4.22 [2.3, 6.1], (2–6)	165
Pace of change (days), mean [IC95%], (min–max)	3.50 [1.6, 5.4], (0–5)	137
NPWT side effects^d^, *n* (%)	101 (54)	186
NPWT shutdown for intolerance, *n* (%)	2 (1.1)	186
NPWT shutdown for inefficiency, *n* (%)	0 (0)	186
Other concomitant treatment^e^, *n* (%)	60 (37)	164
Graft take (%), mean [IC 95%], (min–max)	91.6 [89.7, 93.5], (5–100)	161
Full recovery, *n* (%)	110 (79)	140
Time to full recovery (days), mean [IC95%], (min–max)	72.8 [70.9, 74.7], (8–450)	130
Relapse, *n* (%)	59 (62)	95
Duration without recurrence (days), mean [IC95%], (min–max)	197 [195.1, 198.9], (90–450)	4

Abbreviations: CI, confidence interval; F, female; M, male; *n*, number; NPWT, negative pressure wound therapy.

^a^ Trigger factor included: minor or mild trauma, long‐term immobility, phlebitis.

^b^ Skin sample: *Enterococcus*, *Proteus mirabilis*, *Escherichia coli*, *Methicillin‐resistant Staphylococcus*, *Pseudomonas, staphylococcus*.

^c^ Antibiotics prior graft included: Amoxicillin, Cefalexin, Cefazolin, Ciprofloxacin, Levofloxacin, Piperacillin/Tazobactam, Pyosctacine.

^d^ TPN side effects included: cutaneous damage secondary to therapy, eczema, erysipelas, haemorrhagic necrosis, noise‐induced sleep disorders, pain, septic arthritis with *Escherichia coli* of the knee on the same side as the grafted area, slippage of the grafts, wound infection.

^e^ Other concomitant treatment included: Clobetasol propionate, compressive dressing with bandage, dietary counselling, Dapsone, Infliximab, Iloprost, Methotrexate, Methylprednisolone, Mycophenolate mofetil, parathyroidectomy, Pentoxyphylline, plaster splint, Prednisolone, Simvastatin, Tacrolimus.

The underlying vascular condition was compromised in the majority of cases: 70% of patients had venous disease, and 21% had arterial disease. Bacterial cultures were performed on wound samples in 54% of patients, with *Pseudomonas aeruginosa* and *Staphylococcus aureus* being the most frequently identified pathogens. Antibiotic therapy was administered prior to grafting in 28% of cases, predominantly penicillins or quinolones. The mean wound duration was 27.01 months, although 43% of wounds had persisted for less than 1 year. Only 1.6% of patients had a history of skin grafting, 54% had previously undergone NPWT for a mean duration of 6.8 days, and 51% had undergone surgical debridement prior to grafting.

In most cases, split‐thickness skin grafts (STSGs) were harvested from the patient's thigh using a surgical blade or dermatome. The grafts were secured using skin staples or sutures. The mean pressure applied during NPWT was 104 mmHg, with a mean duration of 6.1 days. The mean time to the first dressing change was 4.2 days, with subsequent changes occurring every 3.5 days. The most commonly used interface between the graft and NPWT was black polyurethane foam, although antimicrobial‐impregnated dressings, alginate sheets, betadine gauze, paraffin gauze, silicone gauze and white polyurethane foam were also utilized. Adverse effects related to NPWT were reported in 54% of cases, primarily pain or noise‐related discomfort; infections were rarely reported. NPWT was discontinued in two patients due to adverse effects: one due to pain and the other due to erysipelas.^29^

Concomitant treatments were employed in 37% of cases, including immunosuppressive therapy for pyoderma gangrenosum and compression therapy for venous ulcers. One case of parathyroidectomy was reported for the treatment of calciphylaxis.^12^

The percentage of patients achieving maximal graft take (defined as ≥95%) was 33% (54/162), while 77% (125/162) achieved a graft take of ≥90%. The mean graft take was 92% (Table 1). Complete wound healing was achieved in 79% of patients (110/140), with a mean healing time of 73 days. Complete response without recurrence was observed in 38% of cases (36/95). The mean duration without recurrence, available for four patients, was 197 days.

No patients discontinued NPWT due to inefficacy, and only 1% discontinued due to poor tolerance.

### Exploratory univariate analyses

The results of the exploratory univariate analyses are presented in Table 2a,b. Mean graft take was significantly higher in patients under 65 years of age (95.4%) than those over 65 (88.1%), with a correlation coefficient of −0.395 ([−0.530; −0.240], *p* < 0.001). Graft take was also significantly higher in males than in females (95.1% vs. 88.3%, *p* < 0.001), in patients without arterial disease compared to those with arterial disease (90.4% vs. 83.7%), in patients without diabetes compared to those with diabetes (94.8% vs. 89.9, *p* = 0.021), in patients who had received prior antibiotic therapy compared to those who had not (94.0% vs. 91.1%, *p* < 0.001) and in wounds lasting more than 1 year compared to those lasting less than 1 year (94.9% vs. 88.5%, *p* < 0.001). Graft take also varied significantly by wound type, with higher rates observed in neurotrophic ulcers (99.6%), venous ulcers (92.4%), pressure sores (94.0%), calciphylaxis (95.0%), pyoderma gangrenosum (93.3%) and vasculitis (92.9%) compared to Martorell's ulcers (66.2%), mixed arterial and venous ulcers (86.3%) and arterial ulcers (83.0%) (*p* < 0.001).

**TABLE 2:** Univariate analyses by graft take according to (a) means and medians. (b) correlation coefficients.

(a)	*n*	Min	Max	Mean (IC 95%)	Median [Q25–75]	*p* value
Age						
<65 years	58	20.0	100	95.4 (93.5, 97.3)	100 [90.0–100]	**<0.001**
≥65 years	74	5.00	100	88.1 (86.2, 90)	92.9 [83.0–92.9]	–
Gender						
F	73	5.00	100	88.3 (86.4, 90.2)	92.9 [83.0–100]	**<0.001**
H	59	83.0	100	95.1 (93.2, 97)	100 [92.9–100]	–
Arterial disease						
No	66	5.00	100	90.4 (88.5, 92.3)	95.0 [83.0–100]	**<0.001**
Yes	18	83.0	95.0	83.7 (81.8, 85.6)	83.0 [83.0–83.0]	–
Venous disease						
No	30	20.0	100	88.1 (86.2, 90)	86.5 [83.0–100]	0.54
Yes	63	5.00	100	89.5 (87.6, 91.4)	90.0 [83.0–100]	–
Diabetes						
No	98	5.00	100	89.9 (88, 91.8)	92.9 [83.0–100]	**0.021**
Yes	27	83.0	100	94.8 (92.3, 97.3)	100 [91.5–100]	–
Wound type						
Venous ulcer	82	80.0	100	92.4 (91.1, 93.6)	92.9 [90.0–100]	**<0.001**
Pressure sore	2	88.0	100	94.0 (82.2, 105.7)	94.0 [91.0–97.0]	–
Calciphylaxie	3	90.0	100	95.0 (89.3, 100.6)	95.0 [92.5–97.5]	–
Martorell's ulcer	4	5.00	100	66.2 (22.1, 110.3)	80.0 [46.2–100]	–
Pyoderma gangrenosum	36	20.0	100	93.3 (88.9, 97.6)	94.5 [94.5–100]	–
Neurotrophic ulcer	12	95.0	100	99.6 (98.8, 100.4)	100 [100–100]	–
Arteriel ulcer	13	83.0	83.0	83.0 (83, 83)	83.0 [83.0–83.0]	–
Mixt ulcer	6	83.0	92.9	86.3 (82.2, 90.3)	83.0 [83.0–90.4]	–
Other (vasculitis)	3	92.9	92.9	92.9 (92.9, 92.9)	92.9 [92.9–92.9]	–
Duration > 1 year						
No	95	5.00	100	88.5 (85.8, 91.1)	92.9 [83.0–94.5]	**<0.001**
Yes	47	80.0	100	94.9 (93.3, 96.4)	95.0 [90.0–100]	–
Localization on leg						
No	3	88.0	100	94.3 (87.5, 101.1)	95.0 [91.5–97.5]	0.61
Yes	158	5.00	100	91.6 (89.8, 93.4)	92.9 [90.0–100]	–
Prior antibiotic therapy						
No	132	20.0	100	91.1 (89.6, 92.6)	92.9 [88.2–94.5]	**<0.001**
Yes	26	5.00	100	94.0 (86.3, 101.6)	100 [100–100]	–
Prior skin grafting						
No	159	5.00	100	91.8 (90.0, 93.5)	92.9 [90.0–100]	0.74
Yes	2	60.0	100	80.0 (40.8, 119.2)	80.0 [70.0–90.0]	–
Prior NPWT						
No	61	5.00	100	92.5 (89.3, 95.7)	92.9 [92.9–100]	0.45
Yes	100	20.0	100	91.1 (89.1, 93.0)	94.5 [83.0–100]	–
Prior treatment						
No	27	75.0	100	95.2 (92.8, 97.6)	100 [90.0–100]	**<0.01**
Yes	97	5.00	100	89.7 (86.9, 92.4)	94.5 [83.0–100]	–
Concomitant treatment						
No	80	83.0	100	92.1 (90.4, 93.8)	90.0 [83.0–100]	0.96
Yes	60	20.0	100	92.0 (89.3, 94.6)	92.9 [90.0–93.4]	–
Pre‐graft debridement						
No	66	5.00	100	91.8 (88.9, 94.7)	92.9 [92.9–94.5]	0.89
Yes	95	20.0	100	91.5 (89.3, 93.7)	90.0 [83.0–100]	–

(b)	*n*	Correlation coefficient (95% CI)	*p*
Age (year)	132	−0.395 (−0.530; −0.240)	**<0.001**
Duration NPWT (days)	141	−0.214 (−0.366; −0.0506)	**0.011**
First opening (days)	140	−0.132 (−0.292; 0.0344)	0.12
Pace of change (days)	112	−0.317 (−0.474; −0.139)	**<0.001**
Pressure set (−mmHg)	111	−0.704 (−0.787; −0.595)	**<0.001**
Size (cm^2^)	114	0.217 (0.0347; 0.386)	**0.02**

*Note*: Bold values were only made to underscore the significant values.

Abbreviations: CI, confidence interval; F, female; M, male; max, maximum; min, minimum; *n*, number; NPWT, negative pressure wound therapy; Y, year.

Graft take was inversely correlated with the duration of NPWT, with a correlation coefficient of −0.241 ([−0.366; −0.0506], *p* = 0.011), and positively correlated with wound size, with a correlation coefficient of 0.217 ([0.0347; 0.386], *p* = 0.02). No significant differences in graft take were observed based on the presence of venous disease, wound location on the lower limb, concomitant treatments, pre‐graft debridement, prior skin grafting or prior NPWT.

## DISCUSSION

Our findings demonstrate that the overall graft take for STSGs bolstered by NPWT is excellent, with more than one‐third of patients achieving maximal graft take (≥95%) and over three‐quarters achieving a graft take of ≥90%. These results are highly encouraging and suggest that NPWT is an effective method for securing skin autografts in chronic ulcers.

These findings align with the previous literature on graft take rates for STSGs with NPWT in acute wounds. Sapino et al.^32^ compared graft take with and without NPWT in patients with vascular, traumatic and post‐surgical ulcers. Their study demonstrated a significantly higher graft take rate in the NPWT group (92%) than the non‐NPWT group (72%). Similarly, Scherer et al.^33^ reported superior outcomes with postoperative NPWT compared to bolster dressings in burn patients and those with traumatic injuries, with a graft take rate of 96% in the NPWT group versus 89% in the non‐NPWT group. However, the small sample sizes, heterogeneity of the studied populations and lack of standardized primary endpoints in these studies limit direct comparison with our findings. Despite these limitations, the collective evidence supports the superior efficacy of NPWT in enhancing STSG outcomes.

Körber et al.^7^ were the first to compare graft take rates in chronic ulcers following STSG with NPWT against a control group. Their results showed a significantly higher graft take rate in the NPWT group (92%) than the non‐NPWT group (67%). The clinical success of skin grafting is influenced by multiple factors, including infection, ischaemia, hematoma, graft immobilization, patient compliance and fibrosis. NPWT mitigates these complications by effectively removing exudate and tissue oedema, immobilizing the graft, reducing shear forces and inhibiting bacterial colonization.^34^, ^35^ Additionally, NPWT reduces the required immobilization period post‐grafting, which typically spans several days.^32^

Our study identifies several factors influencing graft take. Patients under 65 years of age exhibited better graft uptake (Table 2a), potentially due to better nutritional status, fewer comorbidities and reduced polypharmacy, all of which can impede wound healing.

The correlation between prior antibiotic therapy and improved graft take may be attributed to the targeted treatment of pathogens identified through wound cultures. *Pseudomonas aeruginosa* and *Staphylococcus aureus* were the most frequently isolated pathogens. Gilliland et al.^36^ demonstrated that the presence of *P. aeruginosa* in ulcers prior to skin grafting significantly impairs graft take. Høgsberg et al.^37^ further highlighted the detrimental impact of *P. aeruginosa* in chronic venous leg ulcers, leading to partial or complete graft rejection. Their study showed complete healing in 73% of ulcers not colonized by *P. aeruginosa*, compared to only 33% in colonized ulcers.^37^ Our results corroborate these findings, suggesting that the combination of surgical debridement and antibiotic therapy effectively reduces bacterial load and improves graft take.

Our study found no significant difference in graft take when NPWT was applied prior to skin grafting. This may be attributed to the fact that surgical debridement was performed in the majority of cases, which likely achieved similar outcomes to NPWT in terms of preparing the wound bed. NPWT has been shown to improve wound quality prior to grafting,^29^, ^37^ but surgical debridement can achieve comparable results more rapidly. Furthermore, surgical debridement is the preferred method for removing infectious biofilms, particularly those formed by *P. aeruginosa* and *S. aureus*.^37^

The optimal duration of NPWT for skin grafts remains a subject of debate. Our study suggests that shorter durations of NPWT are associated with higher graft take rates. This may be due to the fact that prolonged NPWT is often employed in cases of poor initial graft uptake. However, our findings indicate that the majority of grafts are successfully integrated within the first week, suggesting that extending NPWT beyond this period may not enhance graft survival.

In terms of safety, one serious adverse event was reported: the development of erysipelas on the limb ipsilateral to the graft, leading to NPWT discontinuation.^29^ The most common adverse events were sleep disturbances due to NPWT‐related noise and pain. Overall, NPWT was well‐tolerated, with only 1% of patients discontinuing treatment due to intolerance.

### Limitations

Our review has several limitations. The study is subject to information bias, particularly regarding the subjective assessment of graft take by different practitioners at varying time points. The retrospective nature of some included studies, along with the absence of control groups, limits the strength of our conclusions. Additionally, publication bias may have led to an overestimation of graft take rates. Data on patient nutritional status and the impact of grafting on chronic pain were unavailable, which could have provided further insights.

### Strengths

The strengths of this study include its robust methodology, the inclusion of the largest cohort of patients with chronic wounds treated with STSG and NPWT, and the analysis of potential predictive factors for graft success.

Lastly, prospective comparative studies with large cohorts are needed to evaluate graft take in order to avoid the various biases and identify other predictive factors of graft maximal uptake.

## CONCLUSIONS

Our findings suggest that NPWT facilitates excellent graft performance, with near‐complete graft uptake and minimal complications, demonstrating a favourable safety profile. We identified several subgroups in which NPWT appears to be particularly effective, including venous ulcers, male patients, those under 65 years of age, patients without arterial disease and those who received prior antibiotic therapy. Prolonged use of NPWT does not appear to enhance graft uptake, suggesting that short‐term application of less than 1 week is optimal.

The promising results of this study, along with those of previous research, indicate that NPWT improves graft take, accelerates healing and reduces treatment costs, all while maintaining good patient tolerance. Therefore, NPWT should be considered a preferred method for securing skin grafts in the treatment of chronic ulcers. Further large‐scale prospective comparative studies are necessary to confirm these findings and identify additional predictive factors for optimal graft uptake in chronic wounds.

## AUTHOR CONTRIBUTIONS

M. Gamel: substantial contributions to conception and design, or acquisition of data or analysis and interpretation of data. M. Gael: drafting the article or revising it critically for important intellectual content. A. C. Bursztejn: final approval of the version to be published.

## FUNDING INFORMATION

No funding was received for conducting this study.

## CONFLICT OF INTEREST STATEMENT

We have no conflict of interest to declare.

## ETHICAL APPROVAL

The procedures followed were in accordance with the ethical standards of the responsible committee on human experimentation (institutional or regional) and with the Helsinki Declaration of 1975, as revised in 1983.

## ETHICS STATEMENT

Not applicable.

## Supporting information


Data S1:

## Data Availability

The data that support the findings of this study are available from the corresponding author upon reasonable request.
