# Supplementary material for: Skin graft bolstered by negative pressure therapy in chronic wounds: A systematic review
Source: J Eur Acad Dermatol Venereol. 2025 Aug 27;40(2):296–304. doi: 10.1111/jdv.20808 (PMC12843873; doi:10.1111/jdv.20808)
Supplement: Supplementary file 1 — Data S1: [file JDV-40-296-s001.docx]

**Appendix 1 : Search equation used in PUBMED.**

(("Skin Transplantation"[MeSH Terms]) OR (grafting, skin[Title/Abstract] OR (graftings, skin[Title/Abstract] OR dermatoplasties[Title/Abstract] OR dermatoplasty[Title/Abstract] OR skin grafting[Title/Abstract] OR skin graftings[Title/Abstract] OR skin transplantations[Title/Abstract] OR transplantation, skin[Title/Abstract] OR transplantations, skin[Title/Abstract])) AND ((Negative-Pressure Wound Therap*[Title/Abstract] OR Therap*, Negative-Pressure Wound[Title/Abstract] OR Wound Therap*, Negative-Pressure[Title/Abstract] OR Negative-Pressure Therap*, Topical[Title/Abstract] OR Therap*, Topical Negative-Pressure[Title/Abstract] OR Negative-Pressure Dressings[Title/Abstract] OR Closure*, Vacuum-Assisted[Title/Abstract] OR Dressing*, Negative-Pressure[Title/Abstract] OR Negative Pressure Dressing*[Title/Abstract] OR Vacuum-Assisted Closure*[Title/Abstract] OR Topical Negative Pressure Therap*[Title/Abstract]) OR (Negative-Pressure Wound Therapy[Mesh Terms])))

**Appendix 2 : Search equation used in COCHRANE.**

'vacuum assisted closure'/exp/mj AND 'skin graft'/exp/mj

**Appendix 3 : Search equation used in EMBASE.**

MeSH « skin transplantation » AND « negative-pressure wound therapy »
